# The use of constrained methods to analyze the molecular reactivity and to define a new type of pseudo atoms

**DOI:** 10.1007/s00894-024-06071-3

**Published:** 2024-07-16

**Authors:** Andrés Cedillo, José-Remy Martínez-Aguilar

**Affiliations:** Departamento de Química, Universidad Autónoma Metropolitana - Iztapalapa, Av. Ferrocarril San Rafael Atlixco 186, Iztapalapa, 09310 CDMX México

**Keywords:** Electronic structure, Chemical reactivity, Constrained methods, Hydrogen bonds, Pseudo atoms

## Abstract

**Context:**

Constrained methods in electronic structure methodologies add terms to the variational equations and generate solutions that represent distorted electronic distributions. In some cases, the new solutions can be used to study the chemical reactivity of parts of the molecule. Additionally, this contribution presents the use of population constraints to define pseudo atoms in a molecule. The effects of the pseudo atom on the molecular properties are analyzed. The pseudo atoms are used to simulate the inductive effect of the substituent in a group of carbonyl molecules and their effect on the stability of the complexes between these organic species and one molecule of water. A discussion on the assumptions involved in the present definition of pseudo atoms is also included.

**Method:**

The constrained RHF computations are done in a modified Hartree-Fock code for Gaussian basis sets. The selected basis set is STO-6 G.

**Supplementary Information:**

The online version contains supplementary material available at 10.1007/s00894-024-06071-3.

## Introduction

The behavior of matter at a molecular level is described by quantum theory, by the use of the electronic structure methods [1–3]. Quantum mechanics successfully predicts the properties of the atoms, molecules, and solids at different levels of approximation and provides a reliable tool for the design of new compounds and materials with specific properties. The understanding of the way molecules interact and respond to their chemical environment leads to the current state of the chemical reactivity theory [4–11]. Density functional theory (DFT) [2, 3, 12] has played a very relevant role in the development of the chemical reactivity models.

Most of the electronic structure methods rely on the variational principle, since the ground-state wave function, or electron density, minimizes the electronic energy [1, 2]. The corresponding variational problem usually leads to a non-linear Euler-Lagrange equation or set of equations, which are mainly solved by an iterative process, namely, the self-consistent field procedure (SCF). The use of additional constraints within the minimization process modifies the variational problem and changes the minimal solution. This type of procedure gives rise to the constrained SCF methods [13–21]. The solution of the new Euler-Lagrange equations usually leads to a distorted the electronic distribution of the molecular system. In many cases, the electronic distortion generated by the constraints can be used to analyze the response of a molecule under specific conditions.

An alternative use of the constrained methodology is presented here. The new terms on the Euler-Lagrange, coming from the constraint, modify the properties of one part of the molecule and they are used to define a pseudo atom inside of a molecule. Population constraints polarize the molecule and modify the electronic properties of a part of the molecule. The effects of the pseudo atom in the electronic structure of the chemical species are briefly explored in this contribution. The changes on the constrained atoms are used to simulate the inductive effect of the substituent along a group of related organic molecules. The substituent effect has an influence on the stability of the complexes of these organic species with one molecule of water and they are related to the solvation process. The prediction of the effects on the stability of these complexes is also analyzed here.

The constrained methods are described in the “Constrained methods and the effect of the constraint in the molecular properties” section, as well as in the effect of some types of constraints. The effect of some type of constraints on the chemical reactivity of molecules is also analyzed in the “Constrained methods and the effect of the constraint in the molecular properties” section. The “A model for pseudo atoms from the constrained methodology” section presents the use of constraints to simulate pseudo atoms and the corresponding results. Discussions and conclusions can be found in the last sections of this paper.

## Constrained methods and the effect of the constraint in the molecular properties

Constrained methods have been used for several years in the electronic structure context. The main idea consists of the incorporation of additional constraints into the variational method, which itself is a constrained minimization. The variational method is used to find the ground-state wave function, which minimizes the expectation value of the Hamiltonian operator,

1 $$\begin{aligned} \min _{\left\{ \Psi , \langle \Psi | \Psi \rangle = 1 \right\} } {\langle \Psi \left| \hat{H} \right| \Psi \rangle } = E^\text {gs} = \langle \Psi _\text {gs} \left| \hat{H} \right| \Psi _\text {gs} \rangle \text { .} \end{aligned}$$

Usually, the normalization of the wave function is incorporated into the minimization process by the use of the Lagrange multipliers method. That is,

2 $$\begin{aligned} \min _{\Psi } { \left\{ \langle \Psi \left| \hat{H} \right| \Psi \rangle - E \left[ \langle \Psi | \Psi \rangle - 1 \right] \right\} } \text { .} \end{aligned}$$

In the same way, for methods based on the electron density, the energy functional $E[\rho ]$ is minimized using the normalization of the density, $\int {\rho (\textbf{r})} \text { d}\textbf{r} = N$, as a constraint. Along with this contribution, the discussion is centered around wave function methodologies. However, the extension to density-based methods is straightforward.

When additional constraints are added to the minimization process of Eq. 1, the minimal wave function changes, as well as the energy. Consider the following set of constraints,

3 $$\begin{aligned} \left\{ \langle \Psi \left| \hat{L}_i \right| \Psi \rangle = l_i^\circ , i = 1, \ldots , c \right\} \text { .} \end{aligned}$$

All the constraints are incorporated into the minimization process by the use of the Lagrange multipliers method. Then, the new constrained minimization can be written in the form,

4 $$\begin{aligned} \min _{ \Psi } { \left\{ \langle \Psi \left| \hat{H} \right| \Psi \rangle - \varepsilon \left[ \langle \Psi | \Psi \rangle -1 \right] - \sum _{i=1}^c {\lambda _i \left[ \langle \Psi \left| \hat{L}_i \right| \Psi \rangle - l_i^\circ \right] } \right\} } \text { .} \end{aligned}$$

The corresponding variational equation becomes,

5 $$\begin{aligned} \hat{H}_{\left\{ \lambda _i\right\} }^\text {cons} \Psi _\text {cons} = \left[ \hat{H} - \sum _{i=1}^c {\lambda _i \hat{L}_i } \right] \Psi _\text {cons} = \varepsilon ^\text {cons} \Psi _\text {cons} \text { .} \end{aligned}$$

And, from the variational method, the energy associated with the minimal wave function of Eq. 4 follows the following relation,

6 $$\begin{aligned} \langle \Psi _\text {cons} \left| \hat{H} \right| \Psi _\text {cons} \rangle = E^\text {cons} \ge E^\text {gs} \text { .} \end{aligned}$$

The terms coming from the restrictions given in Eq. 3 can be seen as an additional potential, and its strength is modulated by the value of the Lagrange multipliers, $\left\{ \lambda _i\right\}$. Note that the constrained wave function ($\Psi _\text {cons}$) and the constrained energy ($E^\text {cons}$) also depend on the value of the Lagrange multipliers associated with the constraints. The expectation values of the restrictions of Eq. 3 are evaluated with $\Psi ^\text {cons}$. The target values of the constraints can be reached when the Lagrange multipliers are the solution of the following set of non-linear equations,

7 $$\begin{aligned} \left\{ \langle \Psi ^\text {cons}_{\left\{ \lambda _i\right\} } \left| \hat{L}_i \right| \Psi ^\text {cons}_{\left\{ \lambda _i\right\} } \rangle = l_i^\circ , \quad i = 1, \ldots , c \right\} \text { .} \end{aligned}$$

The most used constraints are those related to the population of an atom or a group of atoms in a molecule [14–18, 20–23]. In general, this type of restriction has a simple form. For example, the Mulliken population of the group of atoms $\mathfrak {C}$ takes the form,

8 $$\begin{aligned} N_\mathfrak {C}^\text {Mull} = \sum _{\mu \in \mathfrak {C}} {\sum _\nu {P_{\mu \nu } S_{\mu \nu }}} \text { ,} \end{aligned}$$

here ${\textbf {P}}$ and ${\textbf {S}}$ are the density and overlap matrices, respectively. As it is well known, the Mulliken model can be only used when the basis-set functions are centered at the atomic nuclei. Other population models, like the Hirshfeld partitioning procedure [24] lead to a restriction of the form

9 $$\begin{aligned} N_\mathfrak {C} = \int {w_\mathfrak {C}(\textbf{r}) \rho (\textbf{r})} \text { d}\textbf{r} \text { ,} \end{aligned}$$

where $w_\mathfrak {C}$ is a weight function with either sharp bounds (like Bader’s Atoms-in-molecules model [25]) or fuzzy ones (as in Hirshfeld scheme [24]). The population constraints give rise to an additional potential that attracts or repels the electrons in the constrained region. The molecular dipole moment has been used as a constraint [23, 26]. In this case, the additional potential represents a uniform electric field. Spin constraints have also been applied [19, 23]. It is important to mention that the total spin operator includes a two-body term, while the population and dipole moment constraints are represented by a one-body operator. In all these cases, the constrained molecule reaches the target value or asymptotically approaches to it.

Consider the electronic structure provided by the Hartree-Fock method. The electronic energy is written as

10 $$\begin{aligned} E^\text {HF} = \tfrac{1}{2} \sum _\mu { \sum _\nu {P_{\mu \nu } \left( H^0_{\mu \nu } + F_{\mu \nu } \right) } } \text { ,} \end{aligned}$$

where ${\textbf {H}}^0$ and ${\textbf {F}}$ are the core-Hamiltonian and Fock matrices, respectively. The core-Hamiltonian matrix contains the kinetic and nuclear attraction integrals and its elements depend on the one-electron molecular integrals. The Fock matrix is given by,

11 $$\begin{aligned} F_{\mu \nu } = H_{\mu \nu }^0 + \sum _{\gamma \eta } { P_{\gamma \eta } \left[ \langle \mu \nu \parallel \gamma \eta \rangle - \langle \mu \nu \parallel \eta \gamma \rangle \right] } \text { .} \end{aligned}$$

In the previous equation, the symbol $\langle \mu \nu \parallel \gamma \eta \rangle$ represents a two-electron Coulomb integral. At this level, the density matrix only depends on the molecular orbital coefficients ${C_{\mu i}}$ of the occupied orbitals,

12 $$\begin{aligned} P_{\mu \nu } = \sum _{i=1}^N {C_{\mu i}^* C_{\nu i}} \text { .} \end{aligned}$$

The molecular orbital coefficients minimize the energy, Eq. 10, with the orthonormality restrictions,

13 $$\begin{aligned} \min _{\{ C_{\mu i}\}} { \left[ E^\text {HF} - \sum _{ij} { \varepsilon _{ij} \left[ \sum _{\mu \nu } { C^*_{\mu i}C_{\nu j} S_{\mu \nu } } - \delta _{ij} \right] } \right] } \text { .} \end{aligned}$$

This procedure leads to the Roothaan equations,

14 $$\begin{aligned} {\textbf {F}} {\textbf {C}} = {\textbf {S}} \varvec{\varepsilon }{\textbf {C}} \text { .} \end{aligned}$$

Now, the energy is minimized with a restriction in the electron population of the group of atoms $\mathfrak C$. For the Mulliken model, this constraint is given by Eq. 8, and it is included in the minimization with the Lagrange multiplier $\lambda$,

15 $$\begin{aligned} \min _{\{ C_{\mu i}\}} { \left\{ E^\text {HF} - \sum _{ij} { \varepsilon _{ij} \left[ \sum _{\mu \nu } { C^*_{\mu i}C_{\nu j} S_{\mu \nu } } - \delta _{ij} \right] } - \lambda \left[ N^\text {Mull}_\mathfrak {C} - n^0_\mathfrak {C} \right] \right\} } \text { .} \end{aligned}$$

Here, $n^0_\mathfrak {C}$ represents the target value of the number of electrons assigned to the atoms included in the group $\mathfrak {C}$. The variational equation associated with the minimization of Eq. 15 is similar to the Hartree-Fock case, but with a modified Fock matrix,

16 $$\begin{aligned} {\textbf {F}}^\text {cons} {\textbf {C}}^\text {cons} = {\textbf {S}} \varvec{\varepsilon }^\text {cons} {\textbf {C}}^\text {cons} \text { .} \end{aligned}$$

The modified Fock matrix takes the form,

17 $$\begin{aligned} {\textbf {F}}^\text {cons} = {\textbf {F}} - \lambda {\textbf {G}}^\mathfrak {C} \text { ,} \end{aligned}$$

where the elements of the matrix ${\textbf {G}}^\mathfrak {C}$ are given by

18 $$\begin{aligned} G^\mathfrak {C}_{\mu \nu } = \tfrac{1}{2} \left[ \delta _{\mathfrak {C},I_{\mu }} + \delta _{\mathfrak {C},I_{\nu }} \right] S_{\mu \nu } \text { ,} \end{aligned}$$

the index $I_{\mu }$ represents the atom where the basis-set function $\chi _{\mu }$ is centered at and $\delta _{\mathfrak {C},I}$ is equal to one when the atom *I* belongs to the group $\mathfrak {C}$, otherwise, it vanishes. Notice that the effect of this additional constraint in the Fock matrix can be seen as an additional potential associated with the atoms included in the group $\mathfrak {C}$. The intensity of the additional potential, or the constraint potential, is modulated by the value of $\lambda$. The constraint potential can be attractive or repulsive according to the sign of the Lagrange multiplier. Furthermore, the solution of Eq. 16 depends on the value of $\lambda$. Therefore, the constrained coefficients and all the molecular properties are also dependent on the Lagrange multiplier.

The constrained methodology described here is implemented in our RHF code. The modified Fock matrix, Eq. 17, is constructed from the standard Fock matrix, and the SCF procedure normally converges in a similar number of steps as the traditional case. In the near future, it will be desirable to implement this methodology in an open electronic structure code. This effort will be especially useful to test the present approach in the Kohn-Sham case.

**Fig. 1:**
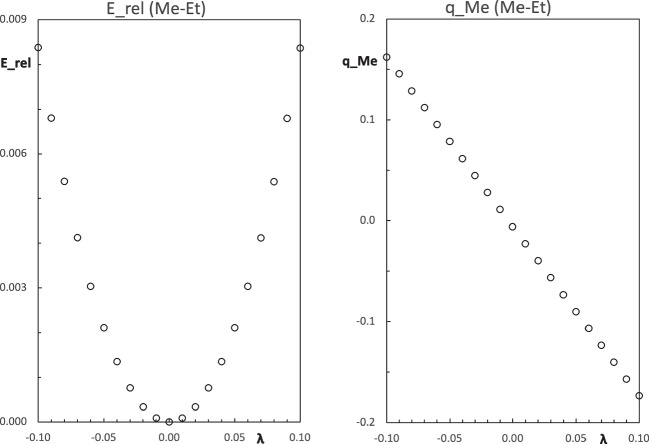
Change of the constrained molecular energy of the propane molecule $\text {CH}_3\text {CH}_2\text {CH}_3$, relative to the unconstrained molecule (left side), and charge of the constrained methyl group as a function of the Lagrange multiplier $\lambda$ (right side). All quantities are in atomic units

As an illustrative example, the electronic structure of the propane molecule, $\text {CH}_3\text {CH}_2\text {CH}_3$, is evaluated with the Mulliken population restriction in the first methyl group. The results computed with the HF/STO-6 G method are shown in Fig. 1 for some values in the Lagrange multiplier in the range $-0.1 \le \lambda \le 0.1$. At $\lambda = 0$, the constrained energy has a minimum and it is equal to the unconstrained case. This is the expected behavior, as it is determined by Eq. 6. The Mulliken charge of the constrained methyl group monotonically decreases with the growth of the Lagrange multiplier $\lambda$. In this small range of variation of $\lambda$, the constrained energy is essentially a quadratic function and the population of the methyl group is almost linear. For a larger variation in the Lagrange multiplier values, these trends are more complex. But, always the minimum of the relative energy is located at the origin and the charge of the methyl group has a decreasing behavior [15, 21, 23].

Propane molecule, like other hydrocarbons, is a non-polar chemical species. The small difference in the electronegativity of the carbon and hydrogen atoms is responsible for the low polarity of the molecule. For the constrained procedure, one of the methyl groups is selected. The rest of the atoms represent an ethyl functional group. Then, the propane molecule has two moieties, Me-Et. In the unconstrained case ($\lambda =0$), the Mulliken charge of the methyl group is quite small, $-0.006$, in agreement with its low polarity. The small negative charge of the methyl group suggests that the methyl group is slightly more electronegative than the ethyl group. For negative values of the Lagrange multiplier, Fig. 1 shows that the charge of the constrained methyl group becomes positive and grows as the value of $\lambda$ is more negative. When the Lagrange multiplier is positive, the charge of the constrained group becomes more negative. This trend shows that the constraint potential repels the electrons in the region of the constrained group when $\lambda <0$, while it attracts them for positive values of $\lambda$. Then, the value of the Lagrange multiplier modulates the electron distribution in the constrained molecule.

In the range of variation of $\lambda$ shown in Fig. 1, it is evident that the constrained energy should also have a quadratic dependence on the charge of the methyl group. The quadratic dependence of the energy with the charge of a fragment of the molecule immediately reminds the Electronegativity Equalization Method (EEM) [27]. Using EEM, it is found that the slope and curvature of the constrained energy as a function of the constrained charge are related to the relative electronegativity and hardnesses of the fragments in the molecule [15, 21, 23].

The response of the chemical species under the new Hamiltonian shows that the population-constraint potential modifies the nature of a functional group within the molecule. The constrained functional group behaves as a better electron-withdrawing or a better electron donor, depending on the value of the Lagrange multiplier $\lambda$. Figure 1 shows that for negative values of $\lambda$ the methyl group becomes positively charged and donates electrons to the ethyl group. That is, the methyl group becomes a better electron donor when $\lambda <0$. On the other hand, for positive values of $\lambda$, the methyl group is a stronger electron-withdrawing group. This type of electronic behavior can be used to simulate the inductive effect produced by a substituent within a molecule. When this kind of constraint potential is applied to a functional group or an atom of the molecule, the constrained group changes its electronic nature and it can be seen as a pseudo group or a pseudo atom. The parameter $\lambda$ modulates the electronegativity of the pseudo group and its effect on some molecular properties is analyzed in the “A model for pseudo atoms from the constrained methodology” section.

## A model for pseudo atoms from the constrained methodology

The use of the constraint associated with the Mulliken population, Eq. 8, on an atom of the molecule modifies the behavior of the constrained atom. The value of the Lagrange multiplier $\lambda$ changes the electronegativity of the constrained atom and the properties of the molecule. The constrained Hamiltonian operator given in Eq. 5 with a population constraint on a particular atom of the molecule defines a new molecular system, namely, the molecule with a pseudo atom. The constrained atom becomes a pseudo atom and its properties are defined by the value of the parameter $\lambda$. Below, the effects of $\lambda$ on the molecular properties are explored for a specific group of molecules. It is well known that the Mulliken populations are very sensitive to the basis set [1]. The tests with different basis sets show that the polarization effects of the constraint potential lead to the same trends as those discussed below.

**Fig. 2:**
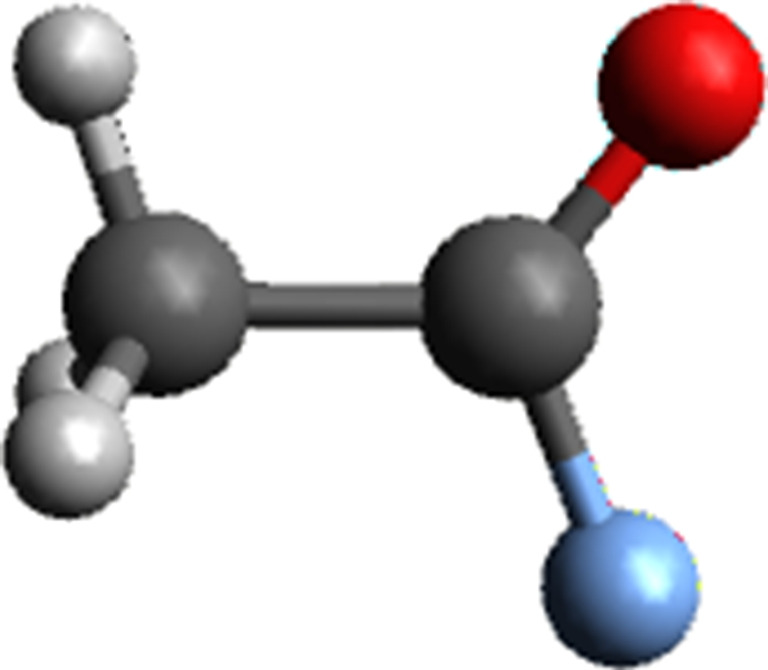
Structure of the carbonyl compounds $\text {Me-C(=O)-R}$, where the substituent $\text {R}$ is represented by a blue sphere

**Table 1:** Functional groups used as substituents in the molecule $\text {Me-C(=O)-R}$

R	Molecule	R	Molecule
$\text {H}$	Acetaldehyde	$\text {CF}_3$	Trifluoroacetone
$\text {CH}_3$	Acetone	$\text {OCH}_3$	Methyl acetate
$\text {NH}_2$	Acetamide	$\text {PH}_2$	Acetyl phosphine
$\text {OH}$	Acetic acid	$\text {SH}$	Thioacetic acid
$\text {F}$	Acetyl fluoride	$\text {Cl}$	Acetyl chloride

Consider a group of molecules with a carbonyl group ($\text {C=O}$) with the formula $\text {Me-C(=O)-R}$, see Fig. 2. The group $\text {R}$ is the substituent and, in this work, it includes the following functional groups: $\text {R = }$ $\text {H}$, $\text {CH}_3$, $\text {NH}_2$, $\text {OH}$, $\text {F}$, $\text {CF}_3$, $\text {OCH}_3$, $\text {Cl}$, $\text {PH}_2$, and $\text {SH}$, see Table 1. The simplest choice for the pseudo atom corresponds to the monoatomic substituents, namely, $\text {R = H, F}$. Therefore, acetaldehyde ($\text {R=H}$) and acetyl fluoride ($\text {R=F}$) are used for population-constrained computations, where the atom at the position $\text {R}$ will be the pseudo atom. The properties of the molecule with the pseudo atom are modulated by the value of the parameter $\lambda$. In general, to avoid any confusion with other atoms in the molecule, the pseudo atom will be labeled with the letter $\text {X}$. The properties of the molecules and the constrained method simulations are computed with the methodology HF/STO-6 G.

**Fig. 3:**
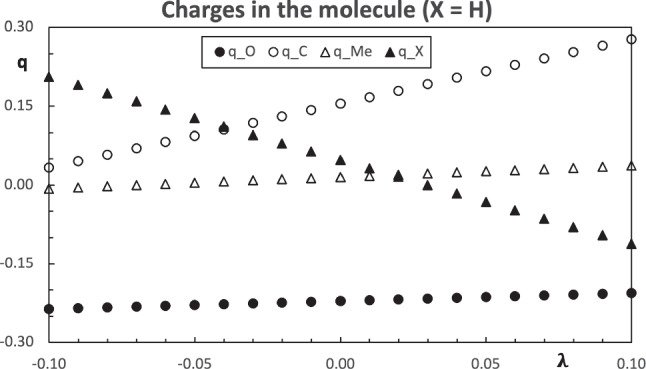
Computed charges of the carbonyl molecule using $\text {X=H}$ as the pseudo atom

**Fig. 4:**
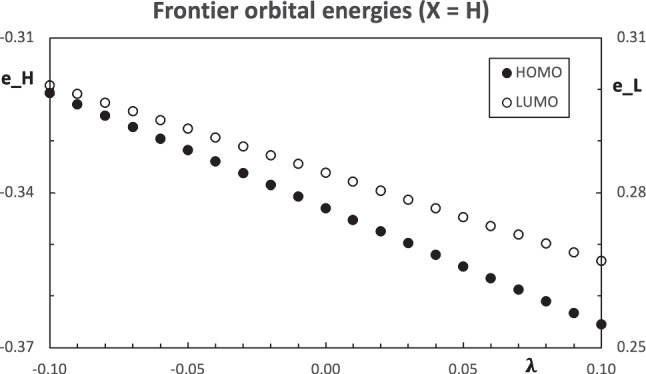
Computed orbital energies of the carbonyl molecule using $\text {X=H}$ as the pseudo atom. All values are in atomic units

**Table 2:** Estimated energy of formation, $E_\text {form}$, of the hydrogen bond stabilized complexes $\text {Me-C(-R)=O}\cdots \text {w}$, and frontier orbital energies, $\varepsilon _\text {HOMO}$ and $\varepsilon _\text {LUMO}$, of the carbonyl molecules

R	$E_\text {form}$	$\varepsilon _\text {HOMO}$	$\varepsilon _\text {LUMO}$	R	$E_\text {form}$	$\varepsilon _\text {HOMO}$	$\varepsilon _\text {LUMO}$
$\text {H}$	$-$0.005920	$-$0.3430	0.2838	$\text {CF}_3$	$-$0.004531	$-$0.3476	0.2411
$\text {CH}_3$	$-$0.006579	$-$0.3276	0.2903	$\text {OCH}_3$	$-$0.006830	$-$0.3458	0.2993
$\text {NH}_2$	$-$0.007555	$-$0.3228	0.3034	$\text {PH}_2$	$-$0.006680	$-$0.2779	0.2881
$\text {OH}$	$-$0.006776	$-$0.3509	0.2971	$\text {SH}$	$-$0.006504	$-$0.2846	0.2891
$\text {F}$	$-$0.005821	$-$0.3721	0.2794				

All the energies are computed with the HF/STO-6 G method and they are reported in atomic units

The computed properties of the molecules with the pseudo atoms are analyzed first. The charges within the molecule with the $\text {X=H}$ as the pseudo atom for different values of the parameter $\lambda$ have a monotone variation and they can be found in Fig. 3. The slope with the larger absolute value corresponds to the constrained atom ($\text {X}$), while the methyl group has the lower variation. Within the carbonyl group, the charge of the carbon atom shows a larger variation than the oxygen. Since the carbon atom is bound to the pseudo atom, this carbon atom is strongly affected by the change in $\lambda$. Aside from the pseudo atom, the carbon atom within the carbonyl group is the atom with the bigger response to the changes in $\text {X}$. The slope of the charges in Fig. 3 is positive for all the fragments of the molecule, except for the pseudo atom, which is negative. Accordingly with Fig. 1, the charge of the constrained group decreases with the increase of the parameter $\lambda$. This is the case for the pseudo atom in the carbonyl molecule. Then, the charge of the rest of the molecule increases. Figure 3 also shows that the closer the other atoms are to the pseudo atom, the larger the inductive effect. The energy of the frontier orbitals also has a monotone change, see Fig. 4. Both, the HOMO and LUMO, decrease their orbital energy as the parameter $\lambda$ grows. This effect is slightly stronger in the HOMO. The growth of $\lambda$ makes the pseudo atom more electronegative. This effect stabilizes the orbital energies and it is similar to the increase in the nuclear charge. When the fluorine atom is used as a pseudo atom, the trend of the changes is similar to the case just discussed.

**Fig. 5:**
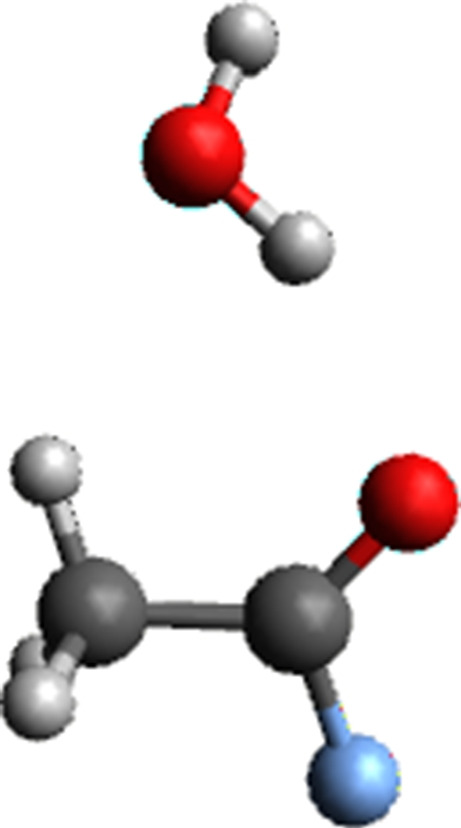
Structure of the hydrogen bond stabilized complex between a carbonyl compound $\text {Me-C(=O)-R}$ and one water molecule. Here, the substituent $\text {R}$ is represented by a blue sphere

Carbonyl molecules ($\text {M}$) are polar chemical species and they have a strong interaction with the water molecule ($\text {w}$). Many of the compounds included in this study are soluble in water or easily react with it. The complex water-carbonyl molecule ($\text {M}\cdots \text {w}$) is stabilized by a hydrogen bond. In the complex, the oxygen atom of the carbonyl group acts as the hydrogen acceptor site, while the water molecule is the hydrogen donor. The strength of the interaction varies with the substituent $\text {R}$ and it is estimated to be around 15 kJ/mol [28]. The geometry of the complexes of the carbonyl species with one molecule of water is optimized at the same computational level. The structure of one of these complexes can be found in Fig. 5. For every complex, the energy of formation is evaluated with the energies of the optimized structures of products and reactants,

19 $$\begin{aligned} E_\text {form}(\text {M}\cdots \text {w}) = E^\text {HF}(\text {M}\cdots \text {w}) - E^\text {HF}(\text {M}) -E^\text {HF}(\text {w}) \text { .} \end{aligned}$$

**Fig. 6:**
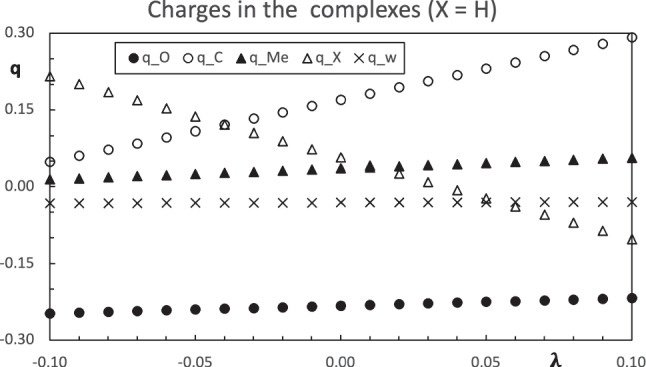
Computed charges within the hydrogen bond stabilized complexes $\text {Me-C(-X)=O}\cdots \text {w}$ using the $\text {X=H}$ as the pseudo atom

Note that the energy of formation defined in Eq. 19 does not include the zero point vibrational contribution. The energy of formation of the complexes $\text {M}\cdots \text {w}$ with all the molecules from Table 1 are collected in Table 2. There is one exception where the hydrogen bond stabilized complex is not formed. The exception corresponds to the acetyl chloride ($\text {R=Cl}$), where the interaction with the water molecule directly leads to the hydrolysis reaction. It is important to mention that the estimation of the energies of reaction with the HF/STO-6 G methodology is not accurate. And, independently of the basis set, the lack of correlation energy in the HF method leads to important errors in the energy of formation. However, similar qualitative trends are observed with other basis sets.

**Fig. 7:**
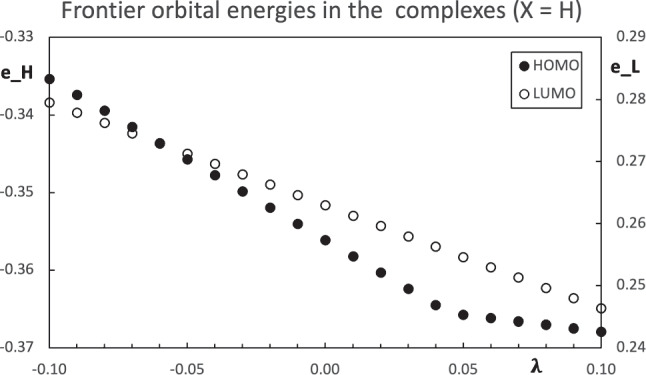
Computed orbital energies for the hydrogen bond stabilized complexes $\text {Me-C(-X)=O}\cdots \text {w}$ using the $\text {X=H}$ as the pseudo atom. All values are in atomic units

The complexes with $\text {R=H and F}$, in their respective optimized geometries, are used with the constrained methodology to simulate the pseudo atom effect for different values of the parameter $\lambda$. The Mulliken charges within the molecule have a similar trend as those in the simulated carbonyl molecules $\text {Me-C(=O)-X}$. The charge of the pseudo atom decreases as $\lambda$ grows, while all the other charges increase their value, see Fig. 6. The effect of the changes in the parameter $\lambda$ decreases with the distance to $\text {X}$. In particular, the charge of the water molecule is practically constant, with variations of a few thousandth of the electron charge. The frontier orbital energies of the simulated complexes decrease with the increase of the parameter $\lambda$, as Fig. 7 shows. However, the HOMO eigenvalues show a different feature, namely, the orbital energy has an abrupt change in the slope. The changes in the parameter $\lambda$ affect in different ways the orbital energies. The change of the slope in Fig. 7 comes from a crossing of the two highest occupied orbitals. For $\lambda < 0.05$, the HOMO is mainly located in the carbonyl group. Since the carbon atom in the carbonyl group is bound to the pseudo atom, its eigenvalue is more sensitive to the variation in $\lambda$ and it strongly stabilizes as $\lambda$ grows. On the other hand, when $\lambda > 0.05$, the HOMO is located at the water molecule and the changes in $\lambda$ have a weaker effect on the corresponding eigenvalue. This behavior is similar to the effect on the charge of the water molecule in the complex, just mentioned above. When two nearby orbitals change at different rates, an orbital crossing can occur and it is observed in Fig. 7. The same trends are found when the fluorine is used as a pseudo atom. For this system, the orbital crossing appears around $\lambda \approx -0.08$.

**Fig. 8:**
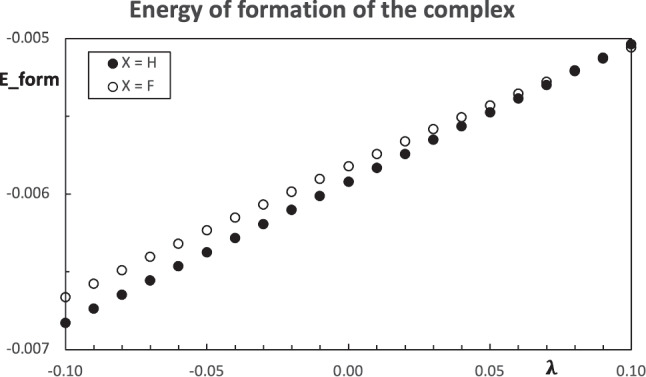
Computed energies of formation of the hydrogen bond stabilized complexes $\text {Me-C(-X)=O}\cdots \text {w}$ using the $\text {X=H,F}$ as the pseudo atom. All values are in atomic units

The energy of formation is also evaluated for the molecules with the pseudo atom. In this case, the geometry of each complex remains fixed while the parameter $\lambda$ changes. The estimated energy of formation of the complexes with the pseudo atoms ($\text {X = H, F}$) are quite similar and they monotonically increase with the growth of the parameter $\lambda$, see Fig. 8. Since the energy of formation, defined in Eq. 19, is negative, the stability of the simulated complex decreases as $\lambda$ grows. Figure 8 also shows a slightly different growth rate for the two pseudo atoms and there is an intersection.

**Fig. 9:**
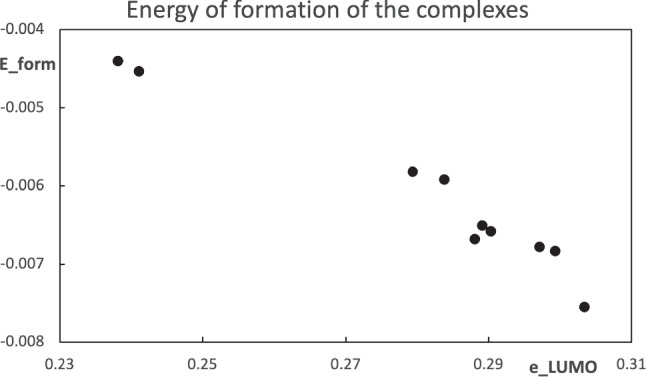
Relation between the estimated energies of formation of the hydrogen bond stabilized complexes $\text {Me-C(-X)=O}\cdots \text {w}$ and the LUMO eigenvalue of the carbonyl molecule. All values are in atomic units

At this moment, there is an important issue to attack. There should be a way to assign a value of the parameter $\lambda$ to each substituent $\text {R}$ in Table 1. Since the inductive effect of the substituent modifies the electron distribution in the molecule, it is important to identify which molecular property of the carbonyl molecule can be used to predict the energy of formation of the hydrogen bond stabilized complexes. An exploration of the computed properties, including Mulliken charges, dipole moment, and frontier orbital energies, unexpectedly led to the LUMO eigenvalue as a predictive property. In the $\text {Me-C(-X)=O}\cdots \text {w}$ complex, with a structure like the one in Fig. 5, the oxygen atom at the carbonyl group and a hydrogen from the water molecule form the hydrogen bond. A small charge transfer from the carbonyl molecule to the water occurs in this interaction, see for example [28]. It is expected that the hydrogen bond should be stronger when the oxygen atom in the carbonyl group is richer in electrons (or a good electron donor). The Mulliken charge of the oxygen atom is not able to correlate with the stability of the complex, nor the other charges in the carbonyl molecule. Since the LUMO eigenvalue provides an approximation to the electron affinity and the carbonyl molecule acts as an electron donor species, its role in the prediction of the stability of the complex was not initially considered. Figure 9 shows the trend between the LUMO eigenvalue of the carbonyl molecule and the energy of formation of the hydrogen bond stabilized complex. From this trend, the value of $\lambda$ that represents the substituent $\text {R}$ comes from the value that matches the LUMO energy, for example, from the data in Fig. 4. Clearly, the value of the parameter $\lambda$ for each substituted molecule will be different if the pseudo atom is modeled by the hydrogen atom ($\text {X=H}$) or the fluorine ($\text {X=F}$). A fit of the LUMO data in Fig. 4 for $\text {X=H}$ allows to get the values of $\lambda$ associated with the LUMO energy of each molecule, and the same is done with the equivalent information of $\text {X=F}$.

**Fig. 10:**
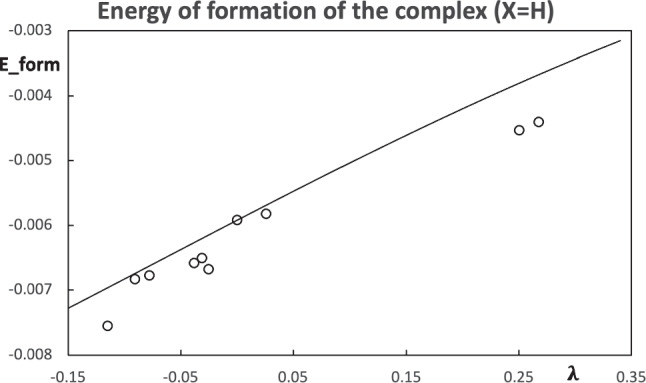
Predicted energies of formation of the hydrogen bond stabilized complexes $\text {Me-C(-X)=O}\cdots \text {w}$ using the $\text {X=H}$ as the pseudo atom (line) and computed values (circles). All values are in atomic units

**Fig. 11:**
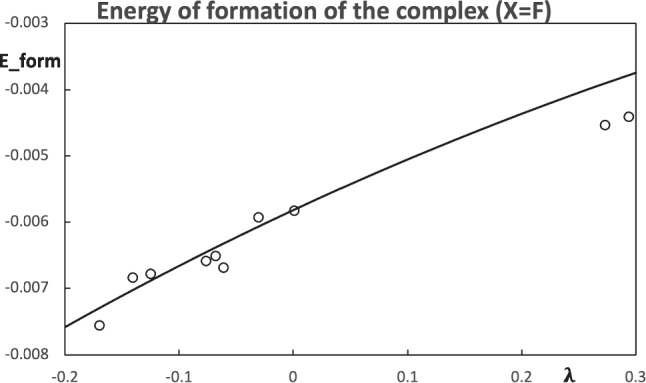
Predicted energies of formation of the hydrogen bond stabilized complexes $\text {Me-C(-X)=O}\cdots \text {w}$ using the $\text {X=F}$ as the pseudo atom (line) and computed values (circles). All values are in atomic units

The comparison between the computed energy of formation of the hydrogen bond stabilized complexes and the estimation from the pseudo atoms can be found in Figs. 10 and 11. The prediction of the energy of formation from the pseudo atoms based on population-constrained methods has the same trend as the computed values. By construction, the constrained atom at $\lambda =0$ is the real atom, and all the molecular properties correspond to the real molecule (in this case, at the HF/STO-6 G level). For this reason, in Figs. 10 and 11, there is a point on the line at $\lambda =0$, which corresponds to the acetaldehyde and acetyl fluoride, respectively. For the two choices of pseudo atoms, the case $\text {X=F}$ provides a better estimation of the stability of the hydrogen bond stabilized complexes.

## Discussion

The constraint potential generated by the Mulliken population restriction provides an alternative model for pseudo atoms within a molecule. This type of constraint transforms an atom into a new electronic center with a tunable electron-withdrawing ability, which is regulated by the value of the parameter $\lambda$. The inductive effect of the substituent is usually associated with its electronegativity [29]. The present work suggests that the population-constrained methodology is an interesting option to simulate the inductive effect. The present results show that the variation of $\lambda$ affects the electronic distribution along the molecule. As expected, the population on the constrained atom shows the largest variation, which is balanced by the redistribution of the rest of the molecule. The redistribution has a stronger effect on the atom that is chemically bound to the pseudo atom. This effect is gradually reduced as the atoms or chemical groups are more separated from the pseudo atom. The strength of the interaction in the complexes mainly depends on the ability of the carbonyl species to form a hydrogen bond with the water molecule. This ability is larger if the electron density is higher at the oxygen atom of the carbonyl group since it directly interacts with the hydrogen of the water molecule. Therefore, a stronger electron-withdrawing pseudo atom depletes the electron density at the oxygen, and the complex becomes less stable. This trend is observed in the constrained computations.

One complex decision is needed. There should be a way to assign the value of the parameter $\lambda$ to each substituent and there is no definitive answer yet. A pragmatic way to attack this problem is the approach chosen in this contribution. The computations of the molecules and complexes show that the LUMO energy of the carbonyl molecules correlates with the energy of the formation of the complexes. From this observation, which needs a deeper analysis, the LUMO energy of the molecules is used to assign a value of $\lambda$ to each substituent. That is, the value of $\lambda$ is chosen to match the LUMO energy of the substituted molecule. The correlation between both quantities leads to a good trend for both choices of the pseudo atom, as discussed above. In future work, the consequences of the way of choosing the values of the parameter $\lambda$ will be analyzed.

## Conclusions

Constrained methods modify the Hamiltonian operator and distort the chemical species in a specific way. The population restriction modifies the electronegativity of the constrained region and polarizes the chemical species. This effect, though the constraint potential, provides a model for pseudo atoms, which is modulated by the parameter $\lambda$. The present model for pseudo atoms requires a deeper analysis to identify its predictive potential. The effect of the basis set and other type of population models are under investigation. The use of other kind of constraints also deserves attention and will be a subject of future scrutiny.

In most of the cases, the application of simple constraints does not require new molecular integrals and its implementation in electronic structure codes is straightforward. Usually, the convergence of the new variational equations is similar to the unconstrained case. For this reason, the simulations with constrained methods do not need a bigger computational effort. The use of other constraints and their possible applications still are an open field of research.

**Supplementary information** The properties of molecules with the pseudo atom $\text {X=F}$, as well as some additional figures, can be found in the supplementary file. The discussions along the text mention that the behavior of the properties with the second choice of a pseudo atom is similar to that shown in the Figures for $\text {X=H}$. This additional material allows the readers to verify the similarities and visualize the small differences for both choices of pseudo atoms.

### Supplementary Information

Below is the link to the electronic supplementary material.

Supplementary file 1 (pdf 867 KB)
