# Supplementary material for: The use of constrained methods to analyze the molecular reactivity and to define a new type of pseudo atoms
Source: J Mol Model. 2024 Jul 16;30(8):269. doi: 10.1007/s00894-024-06071-3 (PMC11252233; doi:10.1007/s00894-024-06071-3)
Supplement: Supplementary file 1 — (pdf 867 KB) [file 894_2024_6071_MOESM1_ESM.pdf]

# Supplementary Information for The use of constrained methods to analyze the molecular reactivity and to define a new type of pseudo atoms.

Andrés Cedillo[1*] and José-Remy Martínez-Aguilar[1†]

[1*]Departamento de Química, Universidad Autónoma Metropolitana - Iztapalapa, Av. Ferrocarril San Rafael Atlixco 186, Iztapalapa, 09310, CDMX, México.

*Corresponding author(s). E-mail(s): cedillo@xanum.uam.mx;
Contributing authors: rmseid99@gmail.com;
†These authors contributed equally to this work.

## List of Figures

1 Computed charge of the pseudo atom in the carbonyl molecules. . . . 2
2 Computed charges of the carbonyl molecule using X=F as the pseudo atom. . . . . . . . . . . . . . . . . . . . . . . . . . . . . . . . . . . 2
3 Computed orbital energies of the carbonyl molecule using X=F as the pseudo atom. All values are in atomic units. . . . . . . . . . . . . . . . . 3
4 Computed charges within the hydrogen bond stabilized complexes Me-C(=O)-X $\cdots$ w using the X=F as the pseudo atom. . . . . . . . . . 3
5 Computed orbital energies for the hydrogen bond stabilized complexes Me-C(=O)-X $\cdots$ w using the X=F as the pseudo atom. All values are in atomic units. . . . . . . . . . . . . . . . . . . . . . . . . . . . . . . . 3
6 Computed orbital energies of the two highest occupied orbitals of the hydrogen bond stabilized complexes Me-C(=O)-X $\cdots$ w using the X=H as the pseudo atom. All values are in atomic units. . . . . . . . . . . . 4
7 Computed orbital energies of the two highest occupied orbitals of the hydrogen bond stabilized complexes Me-C(=O)-X $\cdots$ w using the X=F as the pseudo atom. All values are in atomic units. . . . . . . . . . . . 4

8 LUMO energies of the simulated carbonyl molecules and their linear fit. All values are in atomic units. . . . . . . . . . . . . . . . . . . . . . . . . . 5

Additional plots to the main text. All the computations are done with the HF/STO6-G methodology.

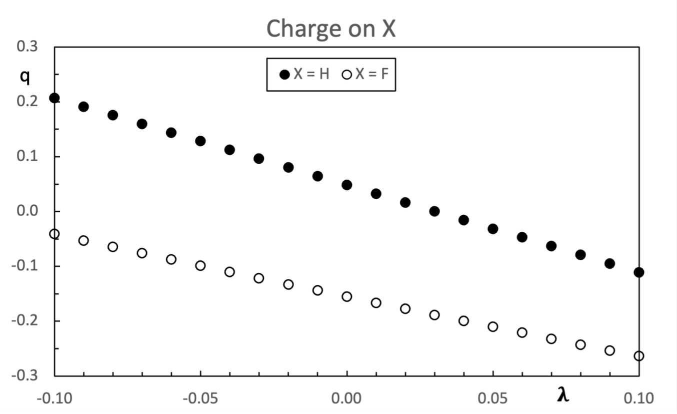


**Fig. 1** Computed charge of the pseudo atom in the carbonyl molecules.

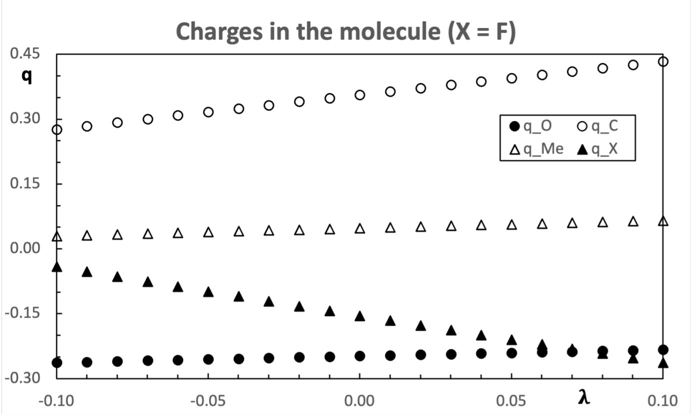


**Fig. 2** Computed charges of the carbonyl molecule using X=F as the pseudo atom.

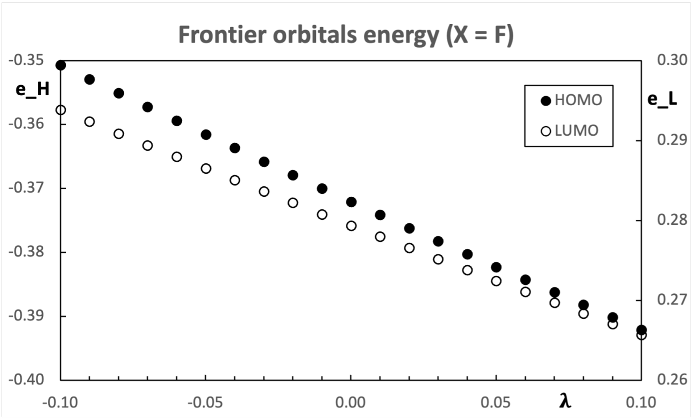


**Fig. 3** Computed orbital energies of the carbonyl molecule using X=F as the pseudo atom. All values are in atomic units.

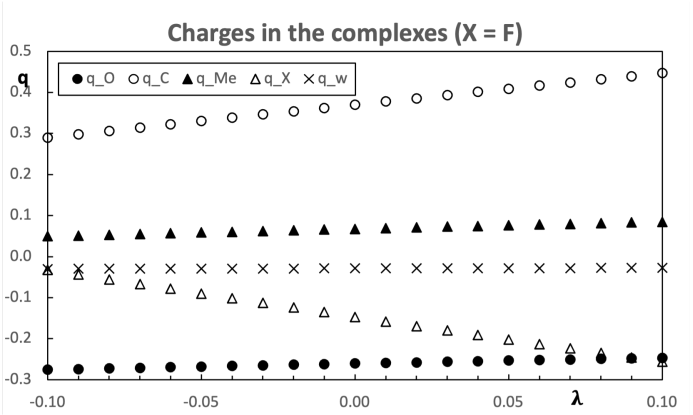


**Fig. 4** Computed charges within the hydrogen bond stabilized complexes Me-C(=O)-X · · · w using the X=F as the pseudo atom.

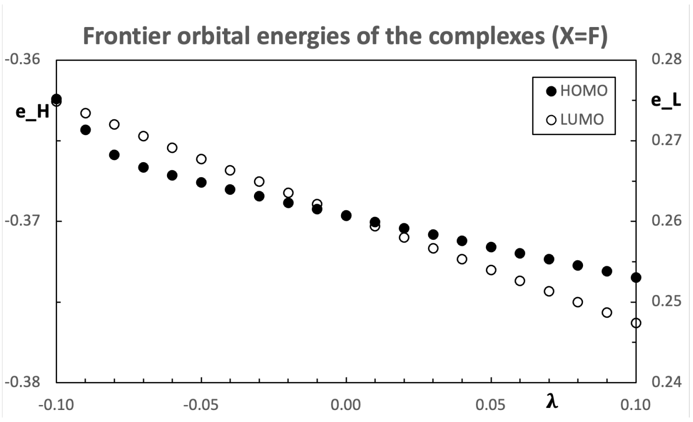


**Fig. 5** Computed orbital energies for the hydrogen bond stabilized complexes Me-C(=O)-X · · · w using the X=F as the pseudo atom. All values are in atomic units.

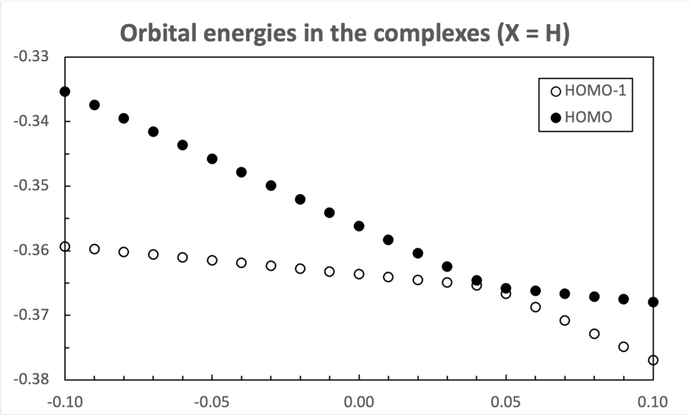


**Fig. 6** Computed orbital energies of the two highest occupied orbitals of the hydrogen bond stabilized complexes Me-C(=O)-X $\cdots$ w using the X=H as the pseudo atom. All values are in atomic units.

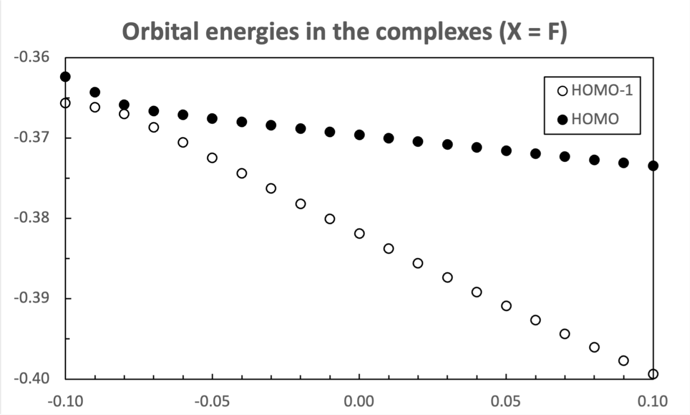


**Fig. 7** Computed orbital energies of the two highest occupied orbitals of the hydrogen bond stabilized complexes Me-C(=O)-X $\cdots$ w using the X=F as the pseudo atom. All values are in atomic units.

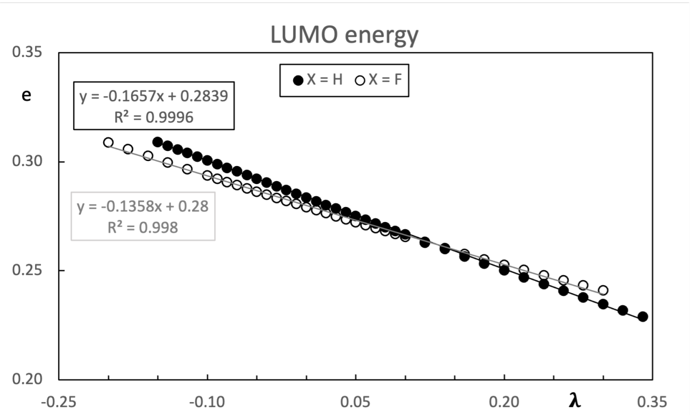


**Fig. 8** LUMO energies of the simulated carbonyl molecules and their linear fit. All values are in atomic units.
